# A functional near-infrared spectroscopy study on hemodynamic changes of patients with prolonged disorders of consciousness responding to different auditory stimuli

**DOI:** 10.1186/s12883-023-03292-6

**Published:** 2023-06-23

**Authors:** Haitao Lu, Jin Jiang, Juanning Si, Yizheng Wang, Fubiao Huang

**Affiliations:** 1 Department of Neurorehabilitation, Beijing Bo’ai Hospital, Rehabilitation Research Center, School of Rehabilitation Medicine, Capital Medical University, Beijing, China; 2 Laboratory of Human Factors Engineering, China Astronaut Research and Training Centre, Beijing, China; 3 School of Instrumentation Science and Opto-electronics Engineering, Beijing Information Science and Technology University, 100192 Beijing, China; 4 Department of Occupational Therapy, Rehabilitation Research Center, School of Rehabilitation Medicine, Beijing Bo’ai Hospital, Capital Medical University, Beijing, China

**Keywords:** Prolonged disorders of consciousness (pDoC), Functional near-infrared spectroscopy (fNIRS), HbO/HbR, Active stimulation, Passive stimulation

## Abstract

Treating prolonged disorders of consciousness (pDoC) is challenging. Thus, accurate assessment of residual consciousness in patients with pDoC is important for the management and recovery of patients. Functional near-infrared spectroscopy (fNIRS) can be used to detect brain activity through changes of oxygenated hemoglobin/deoxygenated hemoglobin (HbO/HbR) concentrations changes and has recently gained increasing attention for its potential applications in assessing residual consciousness. However, the number of fNIRS studies assessing residual awareness in patients with pDoC is still limited. In this study, fNIRS was used to evaluate the brain function in 18 patients with pDoC, including 14 vegetative states (VS) and 4 minimally conscious states (MCS), and 15 healthy controls (HC). All participants accepted two types of external stimuli, i.e., active stimulation (motor imagery, MI) and passive stimulation (subject’s own name, SON). The results showed that the mean concentrations of HbO/HbR in the prefrontal cortex of the HC during the passive stimulation were significantly lower than those of the active stimulation, and the fitting slope was high. However, the hemodynamic responses of the patients with pDoC were opposite to those of the HC. Additionally, the mean concentrations of HbO/HbR increased as the level of consciousness decreased during passive stimulation. Our findings suggest that the residual level of consciousness in pDoC patients can be assessed by measuring brain responses to different stimulations using fNIRS. The present study further demonstrates the feasibility and reliability of fNIRS in assessing residual consciousness in patients with pDoC, providing a basis for its expanded clinical application.

## Introduction

Disorders of consciousness (DoC) arise from severe acquired brain injuries, which cause a continuity of disruption in the arousal and awareness systems of the brain. Common causes of DoC include cerebrovascular disease, craniocerebral trauma, hypoxia, encephalitis, and poisoning. Prolonged disorder of consciousness (pDoC), defined as consciousness impairment lasting for more than 28 days, mainly including vegetative state (VS)/unresponsive wakefulness syndrome (UWS) and minimally conscious state (MCS) [1–3]. Unlike coma (unresponsiveness from which the patient cannot be aroused and in which the patient’s eyes remain closed), patients in VS or MCS are awake. Patients in VS open their eyes spontaneously or in response to stimulation and present preserved autonomic functions, but they are not conscious and show only reflexive behaviors. By contrast, patients in MCS present some repeatable or intentional behaviors but cannot establish functional communication or demonstrate functional object use [4]. A misdiagnosis may lead to mistreatment and cause serious consequences in terms of therapy, prognosis, resource allocation, and ethic issues. Therefore, accurate assessment of residual consciousness is important for managing patients with pDoC.

At present, several evaluation scales, such as the coma recovery scale-revised (CRS-R) [5], the full outline of unresponsiveness (FOUR) [6, 7], music therapy assessment tool for awareness in disorders of consciousness (MATADOC) [8], and sensory modality assessment and rehabilitation techniques (SMART) [8, 9] are commonly used to assess the residual consciousness in patients with pDoC in clinical environment. Nevertheless, it is difficult to obtain appropriate signs/biomarkers from the patients by these scales because some patients with pDoC are in a state of cognitive-motor dissociation (CMD), i.e., command following during functional magnetic resonance imaging (fMRI) and electroencephalogram (EEG) despite being unresponsive at the bedside [3, 10]. Neuroimaging and electrophysiological techniques have significantly facilitated the studies on the evaluation of residual awareness in patients with pDoC [11, 12]. Some studies have shown that fMRI may provide specific cortical features of loss of consciousness, and various dynamic features of EEG signals, and the level of consciousness can be indicated by amplitude and latency through auditory evoked responses, spectral power and signal complexity, and functional connectivity [13]. However, metal implants, critical conditions, the use of anesthetics and the high expense greatly limit the application of fMRI [14]. EEG allows for bedside measurements, but low spatial resolution and craniotomies resulting in highly abnormal recordings, which limit its accessibility [15]. Moreover, in some patients, specific cognitive abilities may be preserved, meaning different approaches and stimuli might elicit more covert awareness. At last, patients with pDoC may fluctuate in alertness, awareness, and attention over time, one test may not reflect the objective state. Therefore, based on the above mentioned issues, accurate assessment of residual consciousness has always been a difficult clinical problem and the misdiagnosis rate was approximately 40% [16–18]. Therefore, various reliable and stable methods are urgently needed to assess residual consciousness in pDoC patients [13, 19, 20].

fNIRS, known as mobile fMRI, is a non-invasive optical neuroimaging tool that can reflect brain activity through responding to the activity of the cerebral cortex by detecting the changes of HbO/HbR concentrations. With the advantages such as portability, compatibility, repeat assessment friendly, movement tolerability, and higher safety, fNIRS has recently gained attention for its potential applications in assessing residual consciousness [21–25]. Though there are some difficulties in improving contact between the probes and the scalp, and getting high quality signal acquisition, fNIRS can play a vital role in identifying in pDoC patients by assessing command-driven brain activity. However, at present, there is lack of applicable and reliable clinic paradigms for evaluating residual cognition of patients with pDoC.

The evaluation of consciousness level is based on the patient’s response to external stimuli. Several studies showed that patients with pDoC respond differently to stimuli, suggesting that the stimulus selection is decisive for accurately evaluating pDoC patient status [13, 14, 25]. Therefore, we hypothesized that the different levels of auditory stimulation may help detect residual consciousness more accurately. In the present study, fNIRS was used to compare the differences in hemodynamics over the prefrontal cortex in response to SON and MI stimuli in pDoC patients as well as HC, to further demonstrate the feasibility and reliability of fNIRS in assessing residual consciousness and to provide an objective bedside assessment for patients with pDoC.

## Materials and methods

### Participants

The participants were recruited from the Department of Neurological Rehabilitation, China Rehabilitation Research Center (Beijing, China) from June 2018 to December 2020. The inclusion criteria were: (1) 18–65 years of age; (2) etiology of stroke, traumatic brain injury (TBI), and anoxic, with a duration of more than one month; (3) normal sleep-wake cycle; (4) diagnosed as VS/UWS or MCS according to the CRS-R scale [26]; (5) intact I-V waves of auditory evoked potentials on at least one side; (6) right-handed; (7) informed consent signed by the legal guardian.

The exclusion criteria were: (1) subjects who have changed their names; (2) epilepsy, sympathetic hyperexcitability, or spasticity without effective control; (3) treatment with hypnotics, antiepileptics, and/or antipsychotics for 2 weeks before the study starts; (4) patients with local skull defects who could not wear the fNIRS cap or could not cooperate with the examination for other reasons.

The CRS-R used to evaluate patients were quality controlled. Two clinicians (with 8 years experienced in the treatment of pDoC) were trained in formal CRS-R evaluation assessed the patients three times and compared the mean scores. If the two scores differ, the two clinicians would jointly perform another test to obtain a uniform CRS-R score.

A total of 18 patients with pDoC (14 males and 4 females, aged 47.83 ± 12.88 years) who met the strict criteria were finally selected to complete the experiment. The causes of pDoC include stroke, traumatic brain injury and anoxic encephalopathy, and the duration of consciousness impairment ranges from 1.7 months to 25.2 months. The clinical characteristics of the patients are illustrated in Table 1. In parallel, HC (7 males and 8 females, aged 25.93 ± 6.62 years) with normal hearing, no neurological disease, and no history of brain injury were selected as the control group.

Written informed consent was obtained from each healthy subject or the patient’s legal guardian for each patient in this study. This study was approved by the Ethics Committee of the China Rehabilitation Research Center (Ethics Number CRRC-IEC-RF-SC-005-01).

**Table 1:** Clinical features of patients with pDoC

	Diagnosis	Gender	Age (years)	Duration of DoC (months)	Etiology	CRS-R
Patient 1	VS	M	57	2.8	Stroke	6
Patient 2	VS	M	55	3.4	TBI	6
Patient 3	VS	M	60	2.3	Anoxic	6
Patient 4	VS	M	64	2.2	Stroke	5
Patient 5	VS	M	47	2.8	Stroke	4
Patient 6	VS	M	41	19.9	Stroke	5
Patient 7	VS	M	60	2.8	Anoxic	5
Patient 8	VS	M	31	25.2	Anoxic	7
Patient 9	VS	F	45	15.5	Anoxic	7
Patient 10	MCS	M	65	4.0	TBI	16
Patient 11	MCS	M	48	1.7	Stroke	14
Patient 12	VS	F	23	7.6	TBI	7
Patient 13	MCS	F	45	6.4	TBI	14
Patient 14	VS	M	44	3.1	TBI	6
Patient 15	VS	M	59	8.2	Stroke	7
Patient 16	VS	F	50	2.3	Stroke	7
Patient 17	VS	M	46	4.1	TBI	5
Patient 18	MCS	M	21	14.2	Anoxic	11

VS, vegetative state; MCS, minimally conscious state; CRS-R, coma recovery scale-revised; TBI, traumatic brain injury

### Study design

**Fig. 1:**
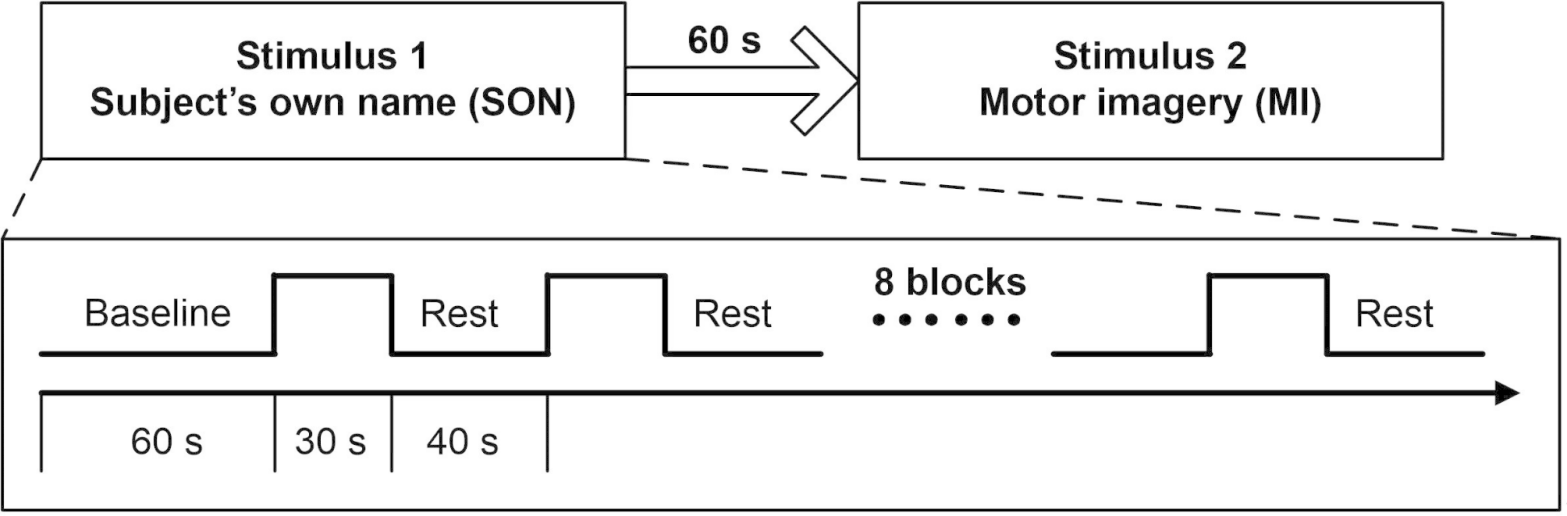
Experimental protocols of participants. BLOCK stimulation was performed for 30 s with an interval of 40 s and repeated 8 times. The two stimuli were presented randomly at an interval of 60 s

Each subject was given two different stimuli: the SON command (“hello, name of the subject”) and the MI command (imagine raising his/her right hand). The stimuli were presented using auditory commands through headphones. For the sound stimuli, words for dubbing were synthesized using iFlytek dubbing software, standard Mandarin male speech, medium speed, 60–70 decibel (dB). The sound was stored in MP3 format, and the stimulation program was presented using EPRIME2.0 software (Psychology Software Tools, Inc., Pittsburgh, PA, USA). The experimental protocol was block-designed. Specifically, the experimental paradigm for each kind of stimulation was consisted of an initial baseline (60 s) followed by 8 blocks. Each block comprised a task period (30 s) and a recovery period (40 s) [27]. The same auditory instructions were repeated 5 times during the SON and MI task period. The 2 stimuli were randomly presented at 60 s intervals. The experimental protocol of this study is shown in Fig. 1.

### Data acquisition and analysis

**Fig. 2:**
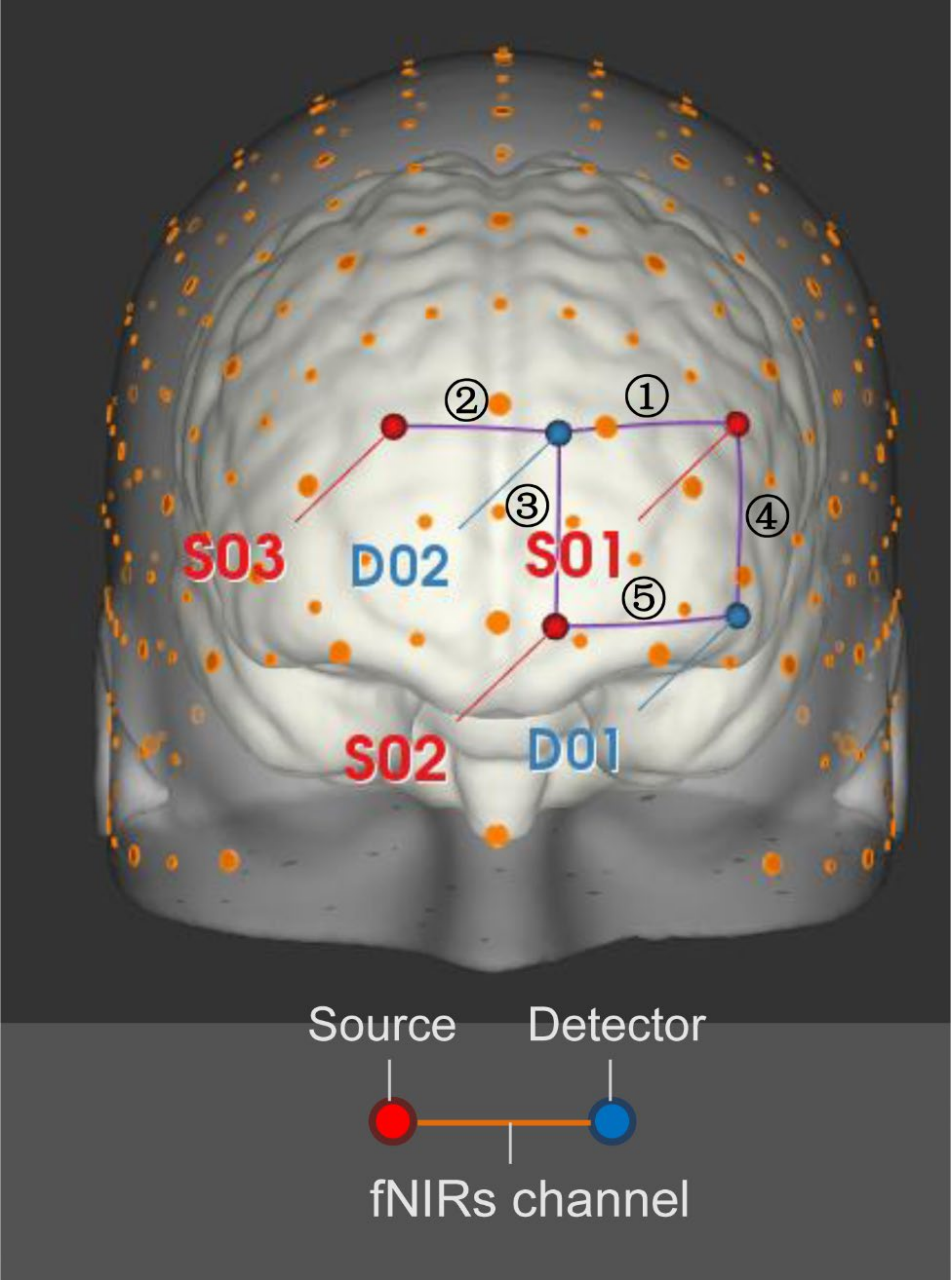
Schematic representing positioning of sources and detectors on the scalp. Three sources (red) and two detectors (blue) optodes were placed on 5 points according to the international 10–20 EEG system. Large orange dots represent reference points of the 10–20 system, whereas small orange dots represent reference points of the extended 10–10 EEG system. The red lines represent source-detector pairs (each forming an fNIRS channel). Image created using NIRSite software

The fNIRS signals of the left prefrontal cortex with 5 channels were measured by the Oxymon MK III (Artinis Medical Systems BV, Nijmegen, The Netherlands) at 760 and 830 nm with a sampling rate of 10 Hz. The fNIRS signals were acquired using three light sources and two receivers, and the optodes were arranged above the prefrontal along the FP1-FP2 line guided by the international 10–20 EEG electrode positions, as shown in Fig. 2. The midpoint between the light sources and the light detectors was placed 3 cm above the center of the upper edge of the left orbital fossa; the distance between the source and the detector pairs was 3 cm.

Data processing was conducted using Homer 2 software and the MATLAB 2017 (The Math Works Inc., Natick, MA, USA). Firstly, the original raw optical data were converted to the relative concentration changes of HbO/HbR based on the modified Beer-Lambert law. Then, the data were band pass filtered between 0.01 and 0.2 Hz to remove cardiac and respiratory interferences. Next, to eliminate motion artifacts in the signal, correlation-based signal improvement (CBSI) was used. CBSI method is a channel-by-channel method used to filter motion artifacts, which is proposed and confirmed by Cui, et al. and Brigadoi, et al. [28, 29], and the trials were rejected by detecting serious motion artifacts which couldn’t be corrected. The data were normalized using the z-score method after pre-processing of HbO/HbR signals [30]. Then, the data of the trials were segmented into epochs, starting 5 s before the stimulation onset and ending 45 s after the audio stimulation. The block-averaged hemodynamic concentration responses and fitting slope of epoch were calculated. The block-averaged mean value of the HbO/HbR concentration reflects the signal amplitude, while the fitting slope indicates the changing rate of the HbO/HbR concentration [31, 32]. Then, the baseline values were subtracted from the data between 3 s before stimulation and 40 s after stimulation, and the brain functional response curves of HbO/HbR signals in the task interval were obtained. The mean concentration was the mean value of the brain functional response curve of HbO/HbR signals from 2 to 20 s after the onset of the task. For the fitting slope of a line of best fit, the linear fitting method was used to obtain the best estimate of the slope of a signal segment, calculated as the fitting slope of the brain functional response curve of HbO/HbR signals from 5 to 15 s after the onset of the task [31].

### Statistical analysis

All data were statistically analyzed using SPSS 22.0 at α = 0.05 significance level. GraphPad Prism 8.0 software was used for statistical charts. The mean ± standard error (SEM) was used for measurement data. The hemodynamic data was divided into three groups (HC, MCS and VS) and were not normally distributed by the Kolmogorov-Smirnov test, so the rank-sum test was used for statistical analysis. The comparisons of the two groups were analyzed by means of the nonparametric Mann-Whitney U test. The comparisons of the three groups were analyzed by means of the Kruskal-Wallis test. A *p*-value < 0.05 was considered statistically significant.

## Results

### The time course of the hemodynamic responses during the SON and MI tasks

**Fig. 3:**
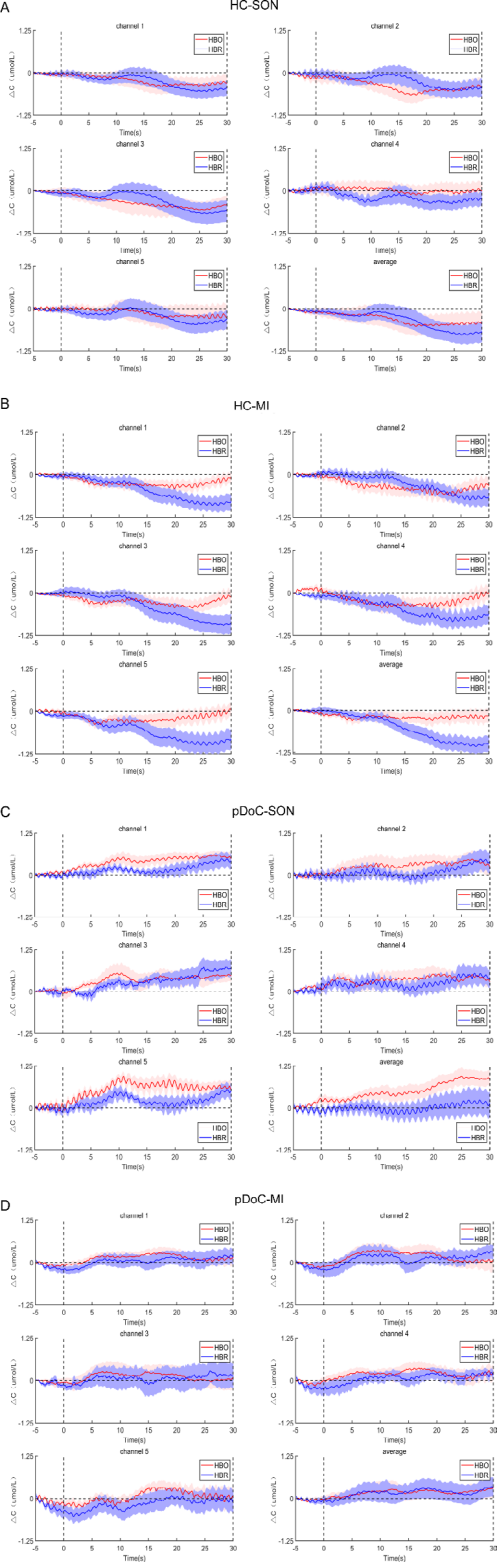
The time course of the group-averaged hemodynamic responses in healthy control and pDoC patients during SON and MI tasks. The time course of mean HbO/HbR in healthy control during SON stimuli (**A**) and MI stimuli (**B**). The time course of mean HbO/HbR in pDoC patients during SON stimuli (**C**) and MI stimuli (**D**)

As shown in Fig. 3A and B, the group-averaged hemodynamic responses of the HC presented the “inverted” pattern (that is negative hemodynamic response) characterized by a decrease in HbO and HbR concentrations. Specifically, for both SON and MI tasks, the hemodynamic responses over the prefrontal cortex were relatively stable during the baseline period. At the task onset, the HbO and HbR concentrations over the prefrontal cortex were decreased.

The group-averaged hemodynamic responses of the pDoC patients were presented different distribution pattern in compared with the HC. Specifically, for both the SON and MI tasks, the hemodynamic responses over the prefrontal cortex were relatively stable during the baseline period. However, after the onset of the task, the changes of the hemodynamic responses of the SON and MI tasks were different from each other. As shown in Fig. 3C, the group-averaged hemodynamic responses of the pDoC patients showed the “typical” hemodynamic response, characterized by an increase in HbO concentration accomplished by a small increase in HbR concentration during the SON task. As shown in Fig. 3D, the hemodynamic responses of group-averaged pDoC patient are characterized by a slightly increase in HbO and HbR concentrations during the MI task.

### Differences in hemodynamic responses between SON and MI stimuli in HC

**Fig. 4:**
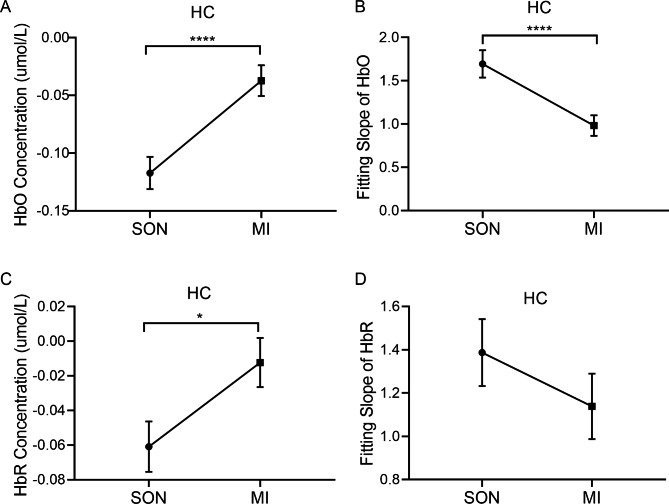
The hemodynamic changes in the healthy control group during SON and MI tasks. The health controls received SON and MI tasks as indicated. The concentrations of HbO (**A**) and HbR (**C**) were recorded. The fitting slope of HbO and HbR are presented in **B** and **D**, respectively. *p < 0.05, ****p < 0.0001

As shown in Fig. 4, the mean HbO/HbR concentrations over the prefrontal cortex of the HC during the MI condition were much higher than after SON stimulation (HbO, Z = -4.345, *p* = 0.000; HbR, Z = -2.241, *p* = 0.025) (Fig. 4A, C). Interestingly, the HbO and HbR concentrations were negative at the group level. In addition, the rates of change in HbO concentrations, namely the fitting slope, were much lower for the MI condition than the SON stimulation (Z = -5.687, *p* = 0.000) (Fig. 4B). In consistent with the trend of HbO, the HbR fitting slope of MI stimulation was much lower than that of SON stimulation, but there was no significant difference (Z = -0.196, *p* = 0.845) (Fig. 4D).

**Fig. 5:**
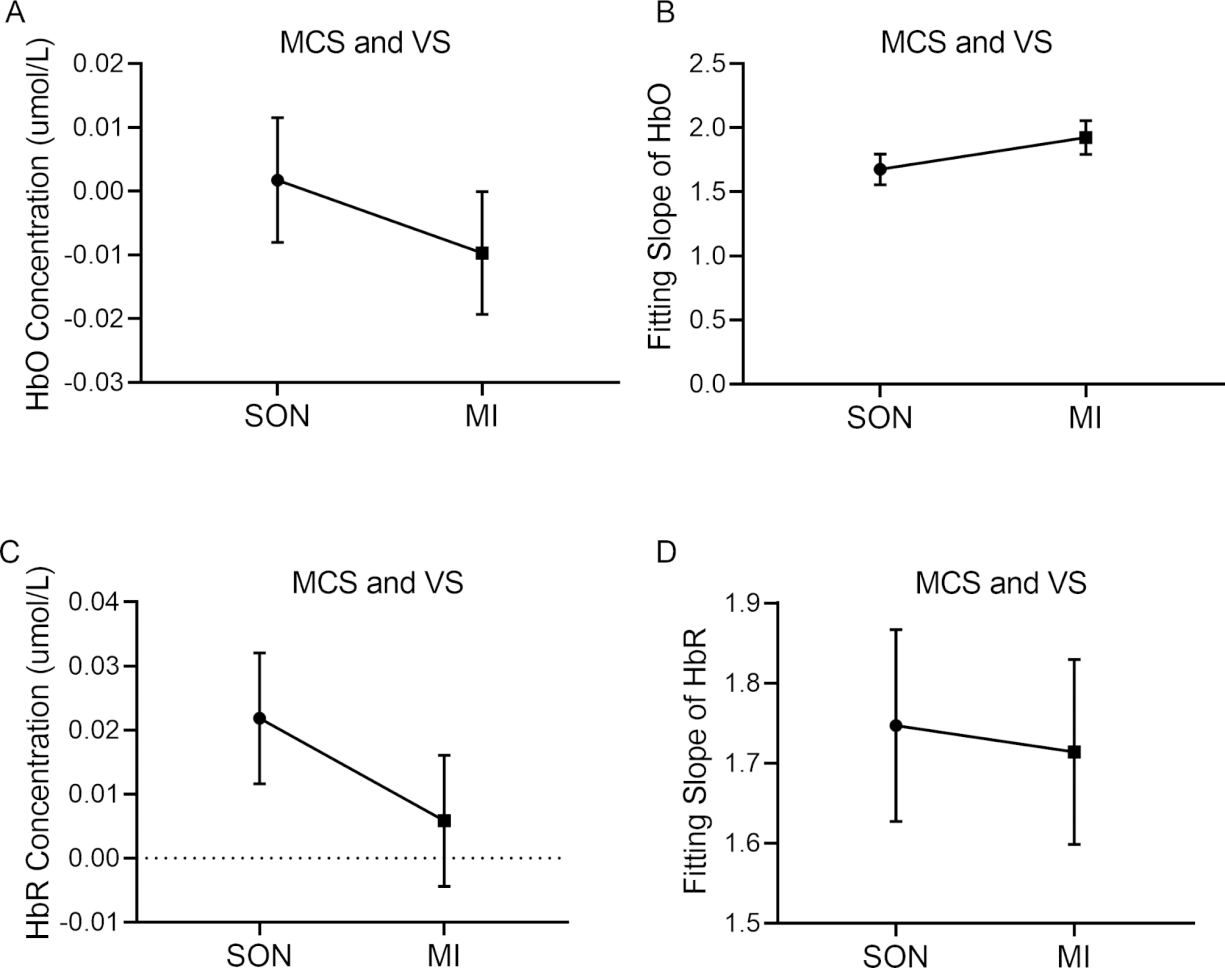
The hemodynamic changes in pDoC patients during SON and MI tasks. pDoC patients, including MCS and VS patients, received SON and MI tasks as indicated. The concentrations of HbO (**A**) and HbR (**C**) were recorded. The fitting slope of HbO and HbR are presented in **B** and **D**, respectively

For the pDoC patients, the changes in the hemodynamic responses were different from those of the HC. Specifically, as shown in Fig. 5, the mean values of the HbO/HbR concentration during the MI condition were significantly lower than after SON stimulation for the pDoC patients. Unlike the HC, the HbO and HbR concentrations of pDoC were positive during the SON tasks (HbO, Z = -0.712, *p* = 0.477; HbR, Z = -1.210, *p* = 0.223) (Fig. 5A, C). The differences in the fitting slopes of HbO/HbR between the two stimuli were not statistically significant (HbO, Z = -1.497, *p* = 0.134; HbR, Z = -0.132, *p* = 0.895) (Fig. 5B, D). Thus, these results indicate that HC and the pDoC patients might present different patterns of hemodynamic responses to the SON and MI stimuli. In addition, the response patterns of pDoC patients were opposed to those of the HC when subjected to SON and MI stimulations.

### The HC and pDoC patients responded differently to the SON stimulation

**Fig. 6:**
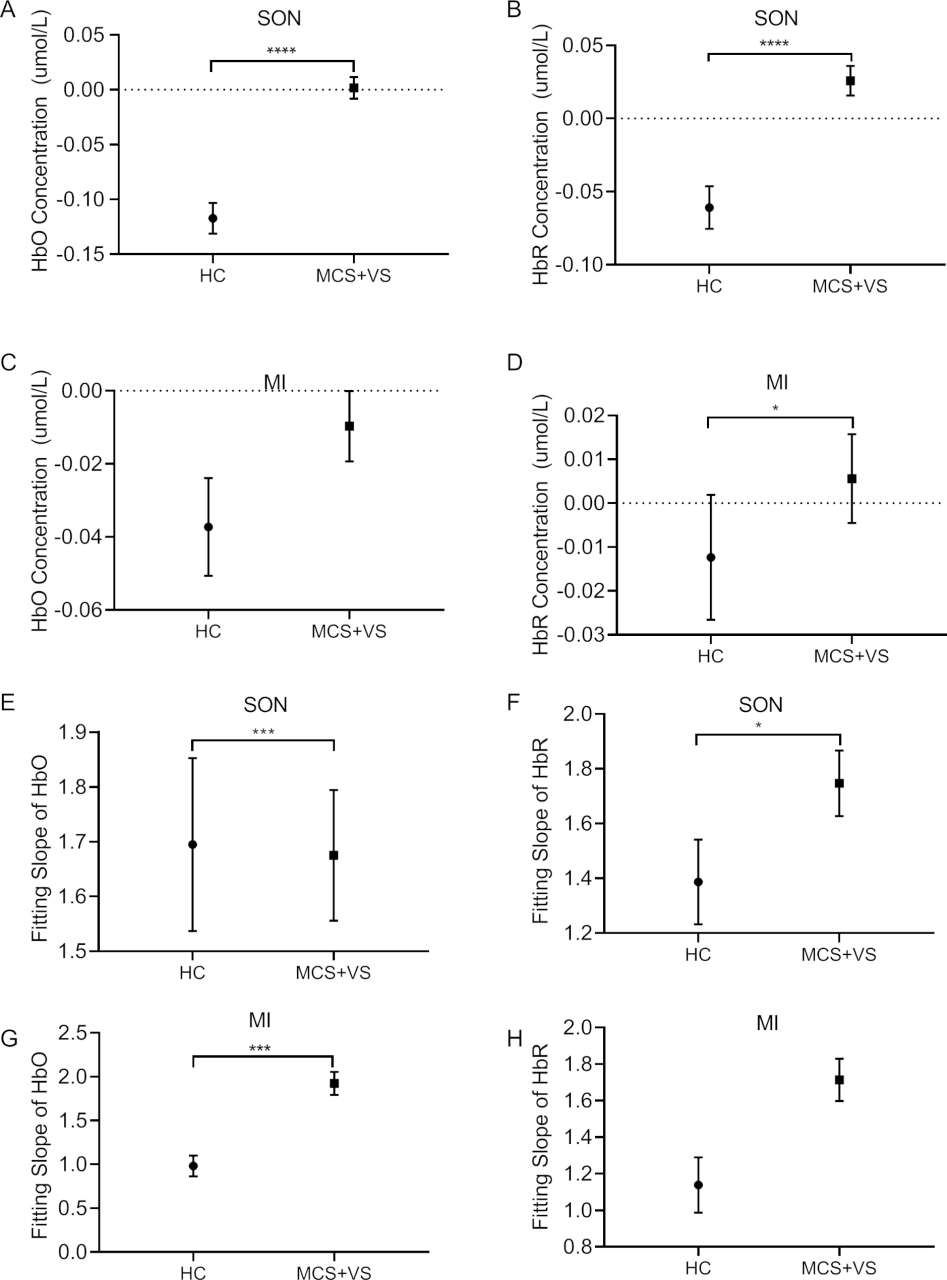
The comparison of hemodynamic changes between the HC and the pDoC patients during SON/MI tasks. The health controls and the patients received SON/MI stimulations as indicated. The HbO/HbR concentrations during SON (**A** and **B**) and MI (**C** and **D**) were recorded. The fitting slopes of HbO/HbR are presented during SON (**E** and **F**) and MI (**G** and **H**). *p < 0.05, ***p < 0.001, ****p < 0.0001

To further determine the differences in the hemodynamic response of the HC and pDoC patients when subjected to the same stimulation, we analyzed the mean HbO/HbR concentrations between the controls and patients. As shown in Fig. 6, the HbO/HbR concentrations of pDoC patients were significantly higher than that of the controls under the SON stimulation (HbO, Z = -8.025 *p* = 0.000; HbR, Z = -3.954, *p* = 0.000) (Fig. 6A, B). The fitting slope of HbO was significantly lower in patients than that of HC (Z = -3.895, *p* = 0.000) (Fig. 6E), whereas the fitting slope of HbR was much higher in patients than in HC (Z = -2.132, *p* = 0.036) (Fig. 6F).

When stimulated by MI, the HbR concentration was significantly higher in pDoC patients (Z = -2.261, *p* = 0.024) (Fig. 6D), and the HbO concentration had the same trend, but it was not significant (Z = -1.934, *p* = 0.052) (Fig. 6C). The fitting slope of HbO in patients was much higher than that of the HC (Z = -3.172 *p* = 0.000) (Fig. 6G). No significant differences were observed in the fitting slope of HbR (Z = -1.559, *p* = 0.119) (Fig. 6H). These data suggested that the task-evoked hemodynamic responses of the pDoC patients were positive activation, while the task-evoked hemodynamic responses of HC were negative activation. Under the MI condition, a strong trend was observed where the concentrations and fitting slope in pDoC patients were higher than in the controls. The results indicate that the pDoC patients have different responses during SON.

### Differences in the hemodynamic responses among the MCS patients, VS patients, and the HC under the SON stimulation

**Fig. 7:**
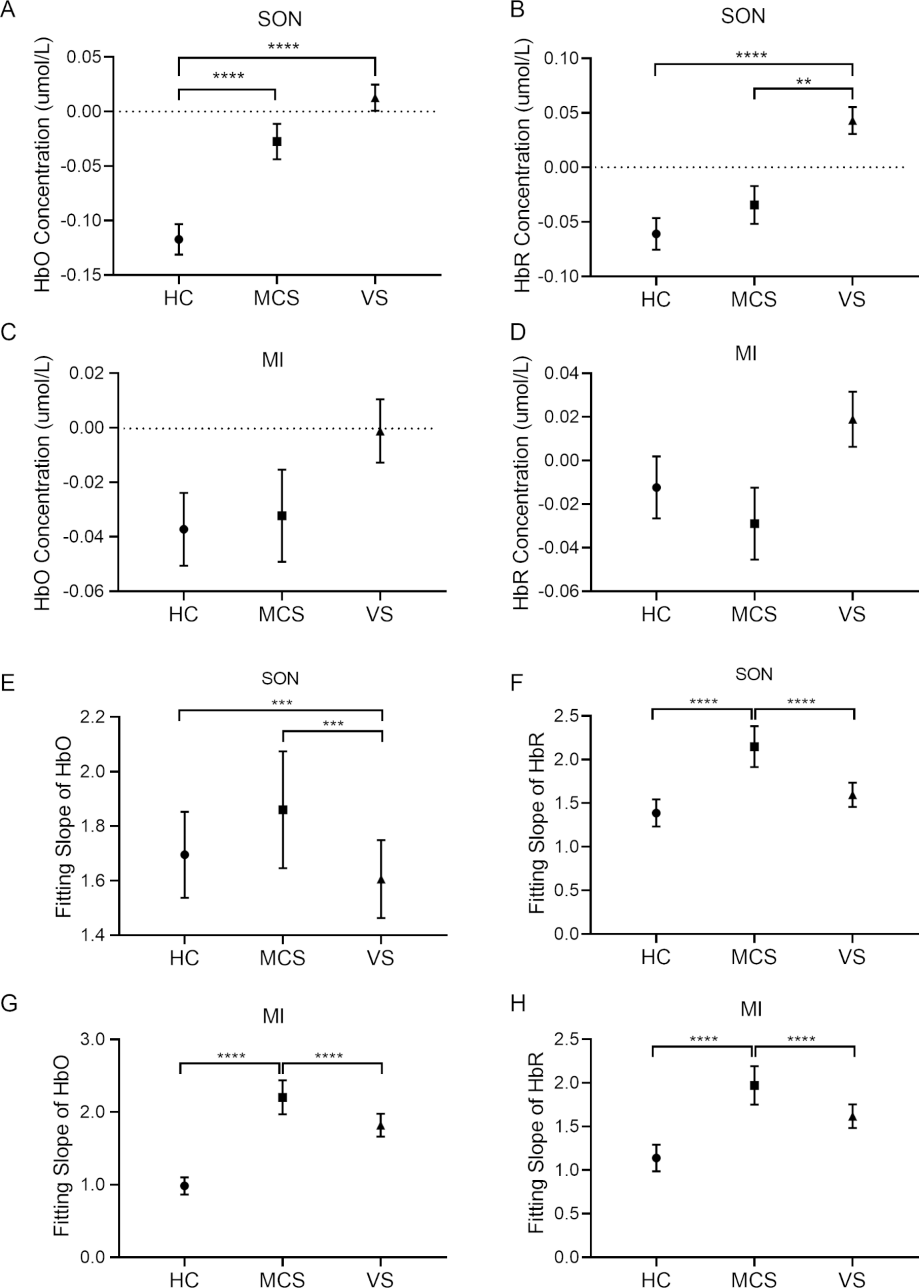
The comparison of hemodynamic changes among HC and the MCS and the VS patients during SON/MI tasks. The health controls and the pDoC patients received SON/MI stimulations as indicated. The HbO/HbR concentrations during SON (**A** and **B**) and MI (**C** and **D**) were recorded. The fitting slopes of HbO/HbR are presented during SON (**E** and **F**) and MI (**G** and **H**). **p < 0.01, ***p < 0.001, ****p < 0.0001

In order to further investigate the differences in hemodynamic responses under the SON and MI tasks, the mean values and the fitting slope of the HbO/HbR concentrations were quantitatively compared among the MCS, VS, and HC. As shown in Fig. 7, under the SON stimulation, the HbO/HbR concentrations of the VS patients were higher than those of the MCS patients. The HbO/HbR concentrations of the MCS patients were higher than those of the HC (HbO, H = 65.38, *p* = 0.000; HbR, H = 30.025, *p* = 0.000) (Fig. 7A, B). The HbO/HbR concentration in HC, MCS, and VS patients went from negative to positive after SON stimulation. The HbO/HbR fitting slope of the MCS patients was higher than that of the other two groups (HbO, H = 40.773, *p* = 0.000; HbR, H = 38.281, *p* = 0.000) (Fig. 7E, F). Under the MI condition, there was a trend that the HbO/HbR concentrations of the VS patients were higher than that of the other groups (HbO, H = 6.018, *p* = 0.052; HbR, H = 6.249, *p* = 0.043) (Fig. 7C, D). The HbO/HbR fitting slope of the MCS was significantly higher than that of the VS patients and the controls (HbO, H = 26.572, *p* = 0.000; HbR, H = 33.648, *p* = 0.000) (Fig. 7G, H). These results may suggest that the VS patients, the MCS patients, and the controls respond differently to SON and MI stimuli.

## Discussion

In the present study, the fNIRS was used to compare the hemodynamic changes of the prefrontal lobe in patients with pDoC and HC in response to different auditory stimuli, including active stimulation (MI) and passive stimulation (SON). The results showed that the hemodynamic responses of the HC group presented negative activations over the prefrontal cortex during both MI and SON conditions at the group level; additionally, the negative activations were more pronounced under SON stimulation. However, the hemodynamic responses of the pDoC patients with different levels showed different distribution patterns during the SON and the MI conditions stimulations compared to HC. Interestingly, the HbO/HbR concentration over the prefrontal cortex changed gradually from negative to positive activation as the level of consciousness decreased.

Active paradigm studies have suggested that although patients with severe brain injury may not have any signs of awareness at the bedside, some of them can even modulate their own brain activity according to commands and occasionally answer yes/no questions by performing MI tasks [32]. EEG and/or fMRI can detect brain activity in approximately 15% of VS patients when stimulated by an active consciousness paradigm, which is indicative of covert cognitive ability [33, 34]. As a complex advanced auditory stimulus, the active stimulation paradigm often employed by DoC patients is MI, which shares a common neural network with motor execution and requires a higher level of consciousness to complete [25]. Increased HbO characterizes a typical MI-evoked hemodynamic response in movement-related regions [35], such as primary motor area, supplementary motor area, and premotor area, no study about prefrontal lobe. However, the sensitivity of detecting covert awareness with active paradigms is low [3], which limits their usefulness. In contrast, SON stimulation causes a more intense clinic response and brain activation in patients because it attaches emotion or some kind of familiarity [36, 37]. Therefore, SON is commonly used as a stimulation mode for detecting residual consciousness in patients with pDoC [38]. Accumulating studies based on neuroimaging and electrophysiological techniques have found that the response to the SON task is evident in the temporoparietal occipital lobe in patients with DoC [19, 38], but no evidence in prefrontal lobe. Therefore, combining the two stimulation modes (SON and MI) and observing the patient’s response pattern to cerebral hemodynamics with two different stimulation levels can improve the accuracy of residual consciousness’s evaluation. Peng Gui et al. [13]. used EEG to detect residual consciousness in DoC patients by auditory hierarchical language processing, and the results showed that the difference in the “residual awareness index” between the MCS and UWS groups progressively increased as the level of language hierarchy increased from rest to words, phrase and sentence conditions. This sets the stage for us to detect residual awareness using different auditory stimuli. The prefrontal cortex, which is an important component of the conscious system, has a causal relationship with conscious perception [27, 39, 40]. In addition, the prefrontal skeleton is relatively intact in most patients with pDoC, and all subjects’ prefrontal skeletons are complete in our study, increasing the study reliability. Moreover, acquiring the hemodynamics over the prefrontal cortex is relatively easy as it is not disturbed by hair and patient’s head/neck position. Therefore, in order to facilitate the clinical application of this experimental paradigm, we examined hemodynamic changes in the prefrontal cortex of subjects.

HbO is an effective physiologic index to describe the oxyhemoglobin content and oxygen molecular energy supply in the brain region, which can indirectly indicate the cerebral cortex’s metabolic and functional activity intensity [41, 42]. In the current study, we found that the prefrontal HbO/HbR was negative in HC under two stimuli (Figs. 3 and 4), which is consistent with previous findings [25, 43]. Holper et al., [43] identified this phenomenon as a decrease in HbO and⁄or an increase in HbR as “inverse oxygenation responses” or “negative activation”, which is often observed during MI tasks or other mental tasks and may be associated with specific tasks or with inhibition of activation in other brain regions. Kempny et al. [25] used motor movement and motor imagery tasks to evaluate consciousness in patients with pDoC and HC. Also, the results showed that negative activations were observed in both groups and tasks in supplementary motor area and primary motor cortex. This result is broadly consistent with our study, although our area of interest is the prefrontal lobe. Eva-Maria, et al. [44] using fNIRS recordings of mental arithmetic got similar results to the present study, i.e. HbO concentration of negative values in HC, while smaller negative values or positive values over anterior prefrontal cortex in the DoC patient. The reasons could be the BOLD response in MRI responding to active inhibition of cortical areas or normal transcallosal inhibition [45]. We also proposed that the lateral areas involved in the tasks might have led to the decrease in the other areas, as the available HbO was delivered to the lateral regions. There may be other reasons we don’t know about until now. So, we plan to add fNIRS optical channels and/or fMRI BOLD signal to explore whole brain responds to get the truth.

Combined with the higher absolute value and slope of HbO concentration change during SON stimulation than MI stimulation, it is inferred that the normal brain responds more strongly to SON stimulation. The present study firstly reported that the absolute values of the hemodynamic activation in HC was significantly higher than those of the pDoC patients during SON stimulation (Fig. 6A and B), and the degree of negative activation decreased or even turned positive with the decrease of the degree of residual consciousness(Fig. 7A and B). The possible reason is that the SON stimulation of normal subjects recruits a certain number of neurons for activation, producing a neurovascular coupling response. Yet, with the decrease in consciousness level, the destruction of consciousness-related neurons and neural networks can only cause a small number of brain responses. Even negative activation is a manifestation of brain activation responses.

However, there are some confusing results in the study. First, we found that when the three groups (HC, MCS, and VS) were compared, the slope of the hemodynamic responses in the MCS group was higher than in the other groups (Fig. 7E and H).In the previous fNIRS studies on pDoC, the mean value of the HbO/HbR concentration is commonly used index, but the fitting slope index is rarely used [31]. Therefore, the number of patients in MCS should be increased for further exploring the reliability of the slope. Second, it is found that for patients with pDoC, the HbO concentration changes in response to SON task were positive, whereas the HbO concentration changes in response to MI task were negative (Figs. 3, 5 and 6). We inferred that the “steal phenomenon” attenuated because of the distance between prefrontal lobe and areas that may be activated, or MI stimulation is more difficult to induce a real response in patients with pDoC. Unfortunately, we cannot conclude whether the measured activation derives from instruction based task performance. So, more homogenous subjects, multiple tasks, repeated examinations, and neuroimaging modalities increase the likelihood of detecting covert awareness in patients with DoC in the future. As an optical tool, fNIRS cannot precisely localize brain regions as fMRI, but this study lays the foundation for further exploration of residual levels of consciousness.

### Limitations and future directions

There are some limitations in the present study. First, the small sample size of subjects may limit the generalizability of the results. Strict inclusion criteria for pDoC patients, such as stable vital signs, minimal head and body movements, and normal cardiopulmonary function, limit the expansion of the sample size in a short term. Because age impacts estimates of hemodynamic responses and optical properties, strict age matching of subjects should be fully considered in future studies. Whether damage and loss of brain structures as well etiology may cause inaccurate results needs to be further clarified. Second, the available optical channels used in this study were limited, and so only the hemodynamics over the left prefrontal cortex was investigated. In further studies, the advanced study design and the improved fNIRS equipment with more optical channels should be used to investigate, so as to accurately evaluate the residual consciousness of patients with pDoC. Moreover, the possible causes of the negative activation should also be explored. In further explorative study, advanced experimental design and improved techniques should be used to comprehensively investigate the residual awareness of the patient with DoC. We hope that, with the accumulation of patients, and the development of neuroimaging techniques and health care, the current findings will be tested soon.

As a pilot fNIRS study, we hope our study could provide useful insights into evaluating residual cognitive ability in this challenging patient population. Additionally, the experimental protocol used in this study has promising clinical utility in the field of DoC.

## Conclusions

The present findings demonstrate that pDoC patients with different residual consciousness present different patterns in hemodynamic response to different auditory stimuli. In addition, the active command-following task and the passive task based on fNIRS provide useful insights for assessing residual awareness in patients with pDoC. In further clinical practice this method could be applied to assist in the identification of patients with locked-in syndrome and DoC. This will further validate the conclusions of this study and await the generation of new findings.

## Data Availability

The raw data used to support the findings of this study are available at https://www.jianguoyun.com/p/DWpZLSsQ_ZWgChiRhKwE (Access Password, fQnKaq) or contact from the corresponding author upon request.
